# Nipah Virus Strain Variation

**DOI:** 10.3201/eid1112.050220

**Published:** 2005-12

**Authors:** Juliet R.C. Pulliam, Hume E. Field, Kevin J. Olival, the Henipavirus Ecology Research Group

**Affiliations:** * Princeton University, Princeton, New Jersey, USA; † Queensland Department of Primary Industries, Brisbane, Queensland, Australia; ‡ Columbia University, New York, New York, USA; § Consortium for Conservation Medicine, New York, New York, USA

**Keywords:** Nipah virus, strain variation, domestic pigs, letter

**To the Editor:** AbuBakar et al. described strain variation in Nipah virus during the 1998–1999 outbreak in Malaysia (*1*). They found an isolate from pigs in Perak, as well as from a flying fox, that differed markedly from pig and human isolates from the main epidemic in southern Malaysia. AbuBakar et al. proposed that this finding indicates 2 separate spillover events from bats to pigs occurred, the first in Perak in 1998 and the second in southern Malaysia in 1999. However, investigations at the time of the outbreak showed that many pigs were moved from Perak onto southern farms in early 1999. We suggest that successive spillovers of the pig population in the north can also explain the observed strain differences between northern and southern isolates.

A model from experimental studies and active farm data demonstrate that Nipah virus may have circulated repeatedly and become endemic within 1 or several large pig farms in Perak (J.R.C. Pulliam, unpub. data), which is consistent with the occurrence of human cases in Perak before the 1998–1999 outbreak. Evolution of the virus population in pigs, followed by the reintroduction in Perak of the original strain from bats and its subsequent southward movement in infected pigs, would explain observed strain differences. Models suggest that evolution of the virus within pig populations would result in lower death rates but prolonged illness. Although the pig-adapted virus strain may have circulated on both northern and southern farms, sampling biases in favor of the more virulent strain would be expected in areas of high death rates, which would explain the observed genetic relationships between sequenced isolates.

We suggest that pigs be experimentally infected with the Perak strain of Nipah virus to determine whether differences exist in illness and death caused by this virus. Further sequencing of virus from archived pig samples will clarify with greater confidence whether multiple strains circulated in both regions.
