# Supplementary material for: Nipah Virus Strain Variation
Source: Emerg Infect Dis. 2005 Dec;11(12):1978–9. doi: 10.3201/eid1112.050220 (PMC3367625; doi:10.3201/eid1112.050220)
Supplement: Related texts

# Reply

**DOI:** 10.3201/eid1112.051102

**Authors:** Sazaly AbuBakar

**Affiliations:** * University Malaya, Kuala Lumpur, Malaysia

**In response:** Pulliam et al. (*1*) presented a model to help explain the observed Nipah virus (NV) strain differences reported earlier by AbuBakar et al. (*2*). The model is built around an assumption that NV was endemic in several pig farms in the north of Malaysia and that a subsequent reintroduction of the original NV caused the fatal encephalitis outbreak in 1998.

While the model is plausible, that NV infection was endemic among pigs before the 1998 outbreak is difficult to imagine in the absence of verifiable evidence. As with any virus that crosses species, NV would likely have caused severe infection, and what happened in 1998 is a classic example. Before NV could have evolved, become less virulent, and subsequently become endemic, it would have been first introduced to pigs. This initial introduction would have caused an outbreak, but no such outbreaks were reported before 1998. Furthermore, the life span of pigs reared in farms is relatively short before they are sent to the markets, which limits the time in which NV evolution could take place. Slaughtering these pigs would also have caused infection among abattoir workers and pork handlers. At present, the finding of 2 different NV strains from 2 different outbreak foci favors the suggestion that 2 possibly overlapping NV outbreaks occurred in Malaysia in 1998. Further investigation of NV archived materials would shed further light into the possible origin of NV in the 1998 Malaysia outbreaks.

## References

[1] Pulliam JRC, Field HE, Olival KJ; Henipavirus Ecology Research Group. Nipah virus strain variation. Emerg Infect Dis. 2005;11:1978–9.1648549910.3201/eid1112.050220PMC3367625

[2] AbuBakar S, Chang L-Y, Mohd Ali AR, Sharifah SH, Yusoff K, Zamrod Z. Isolation and molecular identification of Nipah virus from pigs. Emerg Infect Dis. 2004;10:2228–30.1566386910.3201/eid1012.040452PMC3323361
